# Supplementary material for: The causal relationship between the human gut microbiota and pyogenic arthritis: a Mendelian randomization study
Source: Front Cell Infect Microbiol. 2024 Nov 26;14:1452480. doi: 10.3389/fcimb.2024.1452480 (PMC11629706; doi:10.3389/fcimb.2024.1452480)
Supplement: Supplementary file 1 [file DataSheet1.zip › mendelian test/ebi-a-GCST90027510.csv_forest.pdf]

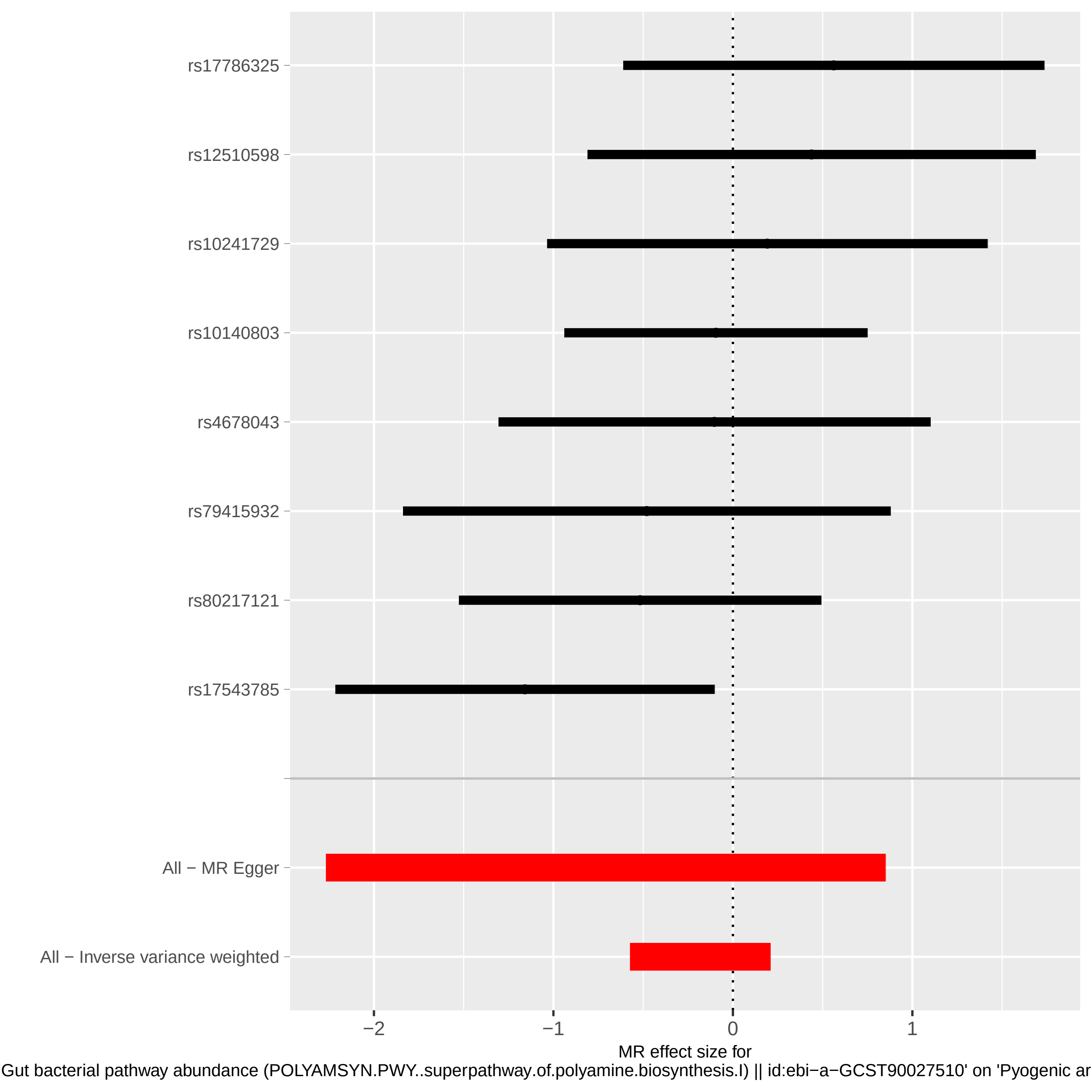

rs17786325
rs12510598
rs10241729
rs10140803
rs4678043
rs79415932
rs80217121
rs17543785
All − MR Egger
All − Inverse variance weighted
−2
−1
0
1
MR effect size for
Gut bacterial pathway abundance (POLYAMSYN.PWY..superpathway.of.polyamine.biosynthesis.I) || id:ebi−a−GCST90027510' on 'Pyogenic ar
